# Targeting WNK1 suppresses acute myeloid leukemia progression and enhances sensitivity to venetoclax

**DOI:** 10.3389/fonc.2026.1828240

**Published:** 2026-06-05

**Authors:** Fangli Chen, Yuanqin Wang, Zhen Fu, Li Chang, Xuewei Jiang, Zhizhi Zhang, Chongyang Li, Ligen Liu, Li Yang

**Affiliations:** 1 Department of Hematology, Tongren Hospital, Shanghai Jiao Tong University School of Medicine, Shanghai, China; 2 Laboratory of Targeted Therapy and Clinical Translation, Shanghai Institute of Hematology, Shanghai Jiao Tong University School of Medicine, Shanghai, China; 3 Department of Medical Oncology, Shanghai Pulmonary Hospital, School of Medicine, Tongji University, Shanghai, China

**Keywords:** acute myeloid leukemia, combination therapy, DNA replication, venetoclax, WNK1

## Abstract

**Introduction:**

The With-No-Lysine (WNK) kinase family plays critical roles in cellular signaling, yet its significance in acute myeloid leukemia (AML) remains unclear.

**Methods:**

We analyzed public databases and primary patient samples for WNK family expression. CRISPR dependency scores were used to assess gene essentiality. The effects of pharmacological WNK1 inhibition (using WNK463 and WNK11) were evaluated on AML cell lines, primary blasts, and mouse xenograft models. Apoptosis was assessed via pro-apoptotic protein expression (Bim, Puma). Transcriptomic analysis identified downstream pathways, and combination studies with venetoclax were performed *in vitro*.

**Results:**

WNK1, but not other WNK family members, was highly expressed in AML, particularly in adverse subtypes such as FLT3-ITD mutated AML. CRISPR screening confirmed WNK1 as essential for AML cell survival. Pharmacological inhibition of WNK1 suppressed proliferation of AML cells and primary blasts, induced apoptosis through upregulation of Bim and Puma, and impeded tumor growth in xenograft models without significant toxicity. Transcriptomic analysis revealed that WNK1 inhibition downregulated DNA replication pathway genes (MCM5, CHAF1B, GINS2), whose high expression correlates with poor prognosis. Elevated WNK1 expression was associated with resistance to venetoclax. Combining WNK463 with venetoclax produced synergistic anti-leukemic effects *in vitro*, accompanied by enhanced Bim upregulation.

**Discussion:**

This study identifies WNK1 as a key oncogenic driver in AML and establishes WNK1 inhibition as a promising therapeutic strategy that not only suppresses AML progression but also sensitizes leukemia cells to venetoclax. These findings provide a rationale for novel combination regimens to overcome drug resistance in AML.

## 1 Introduction

Acute myeloid leukemia (AML) is a highly heterogeneous hematological malignancy, characterized by the clonal proliferation and accumulation of abnormal myeloblasts and other immature myeloid cells in the bone marrow and peripheral blood, and its clinical prognosis varies greatly (1). Although the deepened understanding of the molecular basis of AML has translated into the development of targeted agents against specific mutations like FLT3 (2) and IDH1/2 (3), a substantial proportion of patients continue to confront the formidable challenges of treatment resistance and disease relapse (4). Therefore, there is an urgent need to identify new specific molecular markers to gain a more comprehensive understanding of the molecular pathogenesis of AML, providing new ideas and targets for the development of precise treatment strategies and overcoming drug resistance.

The WNK (With-No-Lysine Kianse) family of serine/threonine protein kinases is characterized by an atypical placement of the catalytic lysine residue, which is crucial for ATP binding in other protein kinases (5). The WNK family in mammals comprises four members, WNK1-4. WNK1 differs from other members of the WNK family in that it is broadly and highly expressed in most animal tissues and cell types, whereas the others show more tissue-restricted expression patterns (6). WNK1 mediates regulatory volume increase (RVI) under hyperosmotic stress by functioning as a molecular crowding sensor. This sensor detects cellular shrinkage and promotes the formation of membrane-less compartments via liquid-liquid phase separation (LLPS) (7). Within these compartments, WNK1 is co-localized with its substrates. The kinase subsequently recognizes and phosphorylates the CCT domain of SPS/Ste20-related proline-alanine-rich kinase (SPAK) and oxidative stress-responsive kinase 1 (OSR1) through its arginine-phenylalanine-any amino acid-valine (RFXV) motif (8). The activated SPAK/OSR1, in turn, phosphorylates and regulates the activity of various ion transporters, including the Na^+^-K^+^-2Cl^-^ cotransporter (NKCC) and the K^+^-Cl^-^ cotransporter (KCC) (8, 9). This phosphorylation cascade constitutes a core mechanism for maintaining renal epithelial ion transport and systemic ion homeostasis. Accordingly, WNK1 is well-established as a key regulator that maintains fluid and electrolyte balance under physiological conditions by modulating ion transporters and potassium channels.

Research on WNK1 has primarily focused on its role in the kidney, where it regulates ion transport and maintains blood pressure and electrolyte homeostasis (10). However, accumulating evidence has established WNK1 as a critical kinase in various cancers. It is frequently overexpressed in tumors and promotes tumor cell growth, survival, and invasion through complex signaling networks, including the MAPK, PI3K/AKT, and WNT/β-catenin pathways (5). Notably, recent investigations have shifted attention to hematological malignancies. In Chronic Lymphocytic Leukemia (CLL), Integrated single-cell genetic and transcriptional analysis suggests WNK1 is a novel driver for CLL progression (11). In multiple myeloma (MM), WNK1 has been identified as a regulator of MYC expression by modulating the IgH enhancer; its inhibition reduces MYC expression and suppresses MM cell growth (12). A latest study revealed that WNK1 was a novel dependency in acute myeloid leukemia (AML) (13). However, two key questions still remain unresolved: the mechanism by which WNK1 regulates AML, and how to harness WNK inhibition for clinical benefit. Therefore, elucidating the molecular mechanisms through which WNK1 contributes to AML pathogenesis will provide a critical theoretical foundation for developing novel targeted or combination therapeutic strategies.

## 2 Materials and methods

### 2.1 Cell lines and patient samples

The acute myeloid leukemia cell lines THP-1, OCI-AML3 and MV4–11 were obtained from DSMZ and maintained in RPMI-1640 medium (Gibco, USA) containing 10% fetal bovine serum (Gibco, USA) and 1% Penicillin-Streptomycin. Cells were cultured at 37 °C in a humidified atmosphere with 5% CO_2_. All cell lines were authenticated by short tandem repeat (STR) profiling. Bone marrow aspirates were obtained from patients with informed consent. Mononuclear cells were subsequently isolated via density gradient centrifugation with Ficoll-Paque Plus (Cytiva), according to the manufacturer’s instructions. The isolated mononuclear cells were frozen in liquid nitrogen or resuspended in complete culture medium for further experiments.

### 2.2 CCK-8 cell viability assay

The cell counting kit-8 (CCK-8) assay was performed to determine cell viability. 2×10^4^ AML cells suspended in complete RPMI-1640 culture medium were seeded into 96-well plates. Various concentrations of drugs were added to each well and incubated for 48h. After incubation, 10 μL CCK-8 reagents (APExBIO) were added into each well and incubated for 2-4h. The optical density (OD) was detected at a wavelength of 450 nm using a microplate reader (Thermo Scientific) and relative growth inhibition rates were calculated.

### 2.3 Apoptosis assay

According to the instructions of the Annexin V-FITC Apoptosis Detection Kit (BD), cells were collected and washed with PBS. Subsequently, cells were incubated with 5 μl Annexin V-FITC and 10 μl PI in the dark for 20 min. Samples were detected by flow cytometry (BD, USA) and analyzed with FlowJo v10.8.1.

### 2.4 Western blotting

Cells were collected and washed twice with cold PBS and suspended with RIPA buffer (Beyotime Biotechnology) containing protease and phosphatase inhibitors (Beyotime Biotechnology). After 30-minute incubation, lysates were centrifuged for 20 minutes and the supernatants were mixed with 5×sample buffer. After incubation at 95 °C for 5 minutes, Protein samples were separated by SDS-PAGE, and transferred to polyvinyl difluoride (PVDF) membranes (Millipore, MA). Membranes were blocked with blocking buffer for 1 h and incubated with the indicated antibodies overnight. After incubation with secondary antibodies, ECL Western Blotting Detection Reagents was used to detect the signal.

### 2.5 Quantitative reverse transcription-polymerase chain reaction

Total RNA was extracted by RNAeasy RNA Isolation Kit (Beyotime Biotechnology) according to the manufacturer’s instructions. cDNA was synthesized with reverse transcription kit (Vazyme) in accordance with the manufacturer’s protocols. The PCR primers used in the study were as follows: *ACTB* (F): CACCATTGGCAATGAGCGGTTC; *ACTB* (R): AGGTCTTTGCGGATGTCCACGT; *WNK1* (F): AGCTGCACCTTTTGGCTCTGAC; *WNK1* (R): GCAGGTAGTGATGTGCAAGAGTC. *MCM5* (F): GACTTACTCGCCGAGGAGACAT; *MCM5* (R): TGCTGCCTTTCCCAGACGTGTA; *CHAF1B* (F): CCTGGAAAAGCCACTCTTGCTG; *CHAF1B* (R): ACAGAAGCACGGAATCCTCCGA; *GINS2* (F): AGCCAAACTCCGAGTGTCTGCT; *GINS2* (R): CTTGTGTGAGGAAAGTCCCGCT. ACTB expression was used as a control for RNA loading. Quantitative PCR reactions were performed using Taq Pro Universal SYBR qPCR Master Mix (Vazyme) on Real-Time PCR System.

### 2.6 RNA-seq

Cell samples were collected and lysed in TRIzol, and total mRNA was then extracted. The mRNA was enriched using mRNA Capture Beads. After purification with beads, the mRNA was fragmented using high temperatures. The fragmented mRNA was then used as a template to synthesize the first strand of cDNA in a reverse transcription enzyme mixture system. While synthesizing the second strand of cDNA, end repair and A-tailing are completed. Next, adapters were ligated, and Hieff NGS^®^ DNA Selection Beads were used for purification to select target fragments. PCR library amplification was then performed, and finally, detection was carried out using the Illumina Novaseq X Plus.

### 2.7 Animal experiment

The BALB/c nude mice (6 weeks old) were housed in an SPF grade environment and subcutaneously inoculated with 5×10^6^ OCI-AML3 cells suspended in 200 μl PBS. Mouse were randomized into vehicle group and WNK463 group when the tumor volume reached 100 mm^3^ (n = 4 mice/group). Vehicle or WNK463 (1.5 mg/kg, intragastric administration, once daily) were given according to the different groups. Tumor volumes were measured every 2 days and body weights were monitored every day. All nude mice were sacrificed after 9 doses of administration. Animal trials were reviewed and approved by the Tongren Hospital Shanghai Jiao Tong University School of Medicine.

### 2.8 Statistical analysis

The relationship between cell viability was analyzed and statistical chats were generated using GraphPad Prism9.0 software. The numerical data were expressed as mean ± standard deviation. Differences between two groups were assessed using Student’s t-test. Multiple comparisons were analyzed using one-way or two-way analysis of variance (ANOVA). Statistical significance was accepted at *P* value < 0.05.

## 3 Results

### 3.1 WNK1 expression was elevated in AML

**Figure 1:**
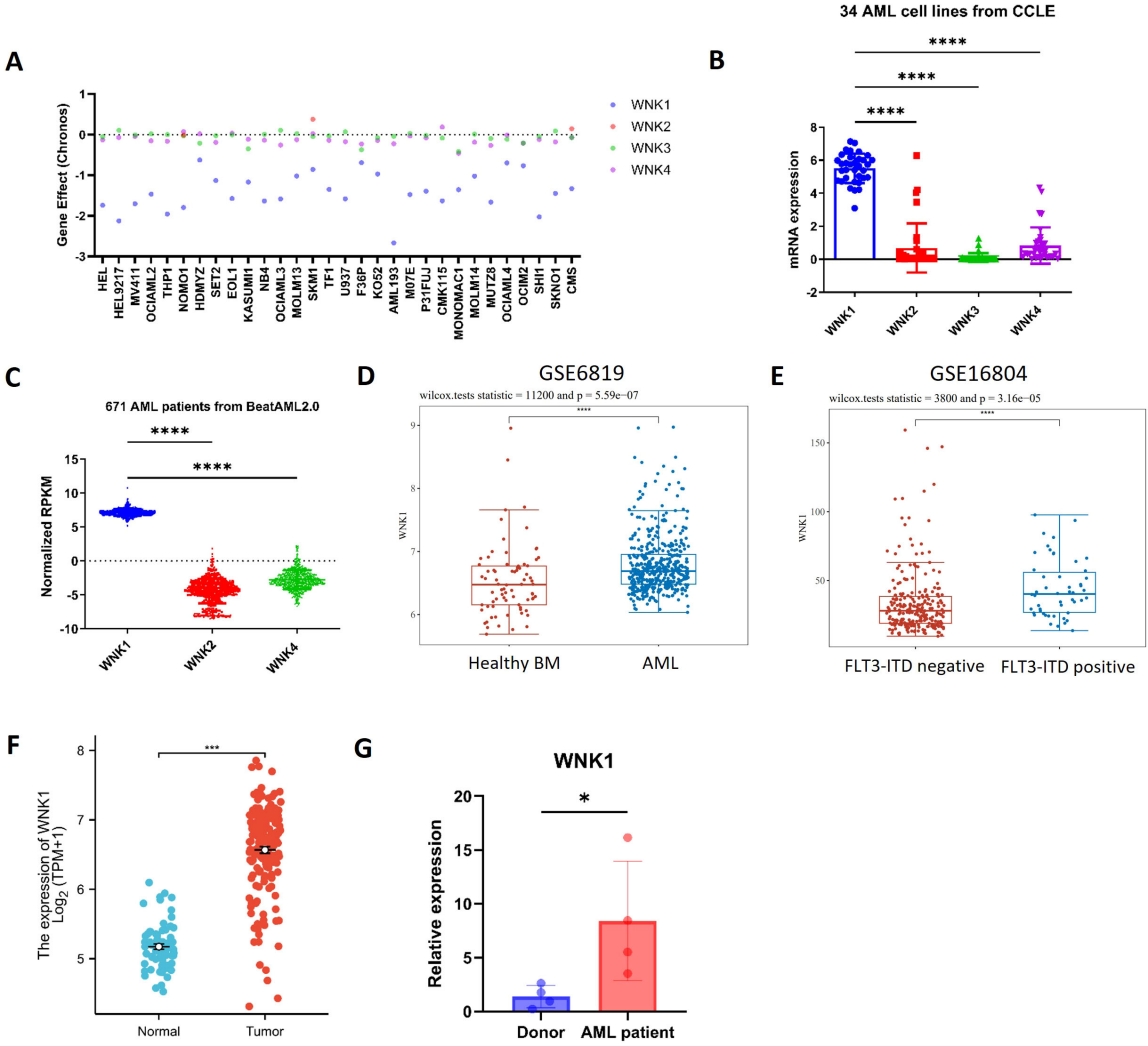
WNK1 is highly expressed in AML. **(A)** The gene effect **(chronos)** of WNK1–4 knockout in various AML cell lines from DEPMAP CRISPR database. **(B)** The mRNA expression of WNK1–4 in AML cell lines (n=34) in Cancer Cell Line Encyclopedia (CCLE) database. **(C)** Normalized mRNA expression of WNK1, WNK2 and WNK4 in primary AML cells in BeatAML2.0 database. **(D)** The mRNA expression of WNK1 in healthy donors (n=76) and AML patients (n=461) in GSE6819 database. **(E)** The mRNA expression of WNK1 in FLT3-WT AML patients (n=243) and FLT3-ITD AML patients (n=50) in GSE16804 database. **(F)** The mRNA expression of WNK1 in AML cells (n=173) in TCGA database and normal tissues (n=70) in UCSC XENA database. **(G)** The qPCR analysis of WNK1 expression in BM mononuclear cells from healthy donors (n=4) and AML (except M3) patients (n=4). *, *P* < 0.05; **, *P* < 0.01; ***, *P* < 0.001; ****, *P* < 0.0001.

To investigated the role of WNK family in AML, we analyzed CRISPR data for 30 AML cell lines in DepMap database. The Chronos scores for WNK1 in all AML cell lines were below 0, strongly suggesting that WNK1 is crucial for AML cell survival (**Figure 1A**). To further evaluate the expression of WNK family in AML, we analyzed WNK expression in 34 AML cell lines from CCLE database. The results indicated a significant elevation of WNK1 mRNA in all AML cell lines, while the expressions of the other WNK family members were relatively low (**Figure 1B**). We confirmed high WNK1 expression in AML patients by analyzing the BeatAML2.0 database across different AML subtypes or specific genetic backgrounds (**Figure 1C**). We also compared WNK1 expression between normal hematopoietic cells and AML cells by analyzing GSE6819 dataset. The results showed AML cells expressed significantly higher levels of WNK1, compared to healthy hematopoietic cells (**Figure 1D**). Interestingly, the result from GSE16804 showed that AML cells with FLT3-ITD mutation expressed higher levels of WNK1 than AML cells with wild type FLT3 (**Figure 1E**). By comparing WNK1 expression using TCGA and UCSC XENA database, we found AML cells expressed higher level of WNK1 than normal cells (**Figure 1F**). We confirmed these results by using quantitative polymerase chain reaction (qPCR) to analyze WNK1 mRNA expression in AML samples from our hospital, which revealed notably higher expression in non-APL AML, compared to healthy donors (**Figure 1G**). Collectively, these findings underscore potential roles of WNK1 in the progression of AML.

### 3.2 WNK1 inhibition suppressed the progression of AML

**Figure 2:**
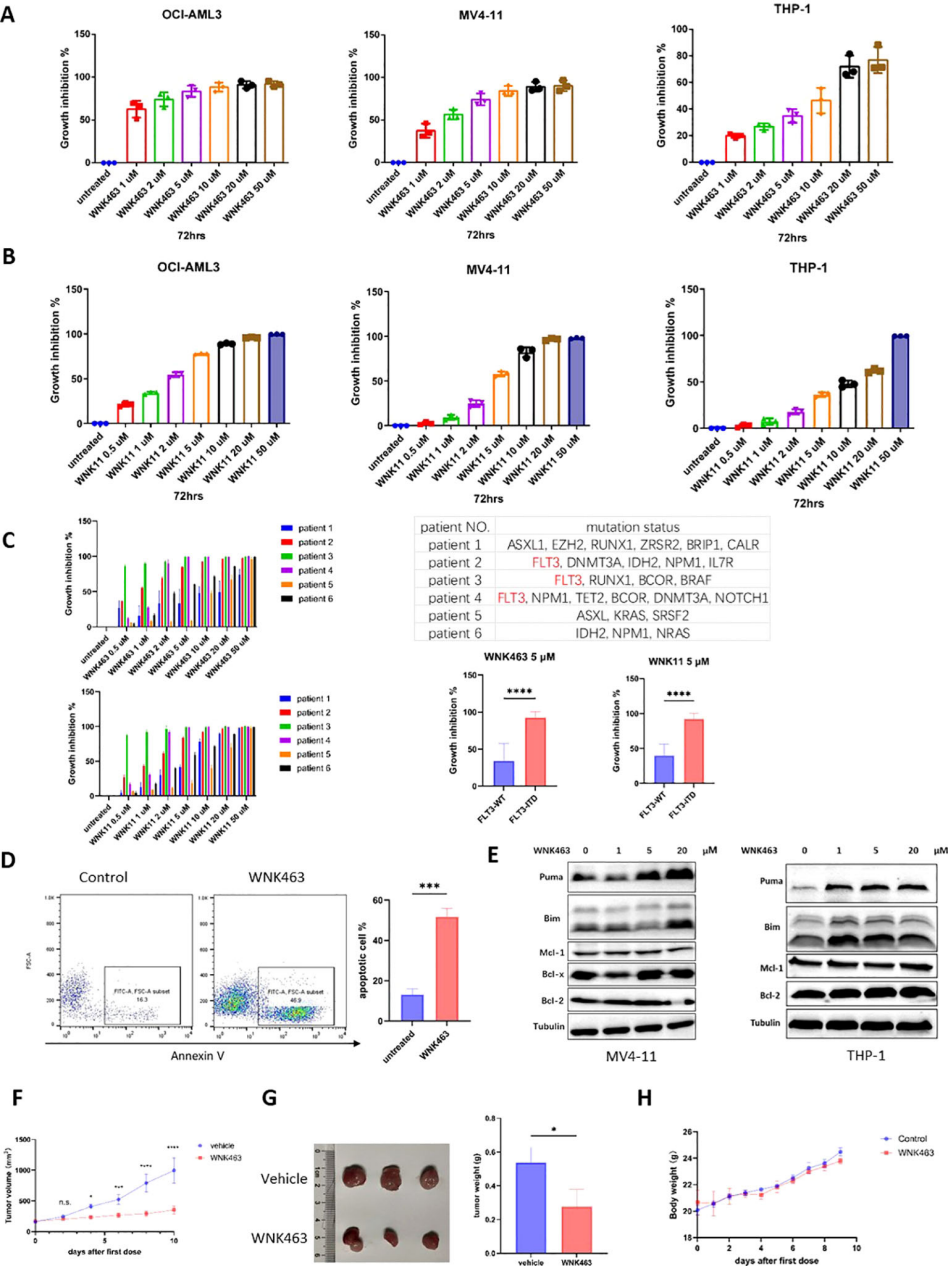
WNK1 is required for survival of AML cells *in vitro* and *in vivo*. **(A, B)** Growth inhibitory effects of WNK463 and WNK11 on OCI-AML3, MV4–11 and THP-1 cells. **(C)** Growth inhibitory effects of increasing concentrations of WNK463 and WNK11 on primary AML cells from 6 patients (mutation status listed in the table). The growth inhibition at 5 μM was compared between FLT3-ITD and FLT3-WT AML cells. **(D)** Schematic diagram of apoptosis in OCI-AML3 cells at 48h after treatment with 0 μM or 5 μM of WNK463 detected by flow cytometry. Apoptosis rates were summarized in statistical histogram. **(E)** Western blot analysis was performed to detect the expression of anti-apoptotic and pro-apoptotic protein in MV4–11 cells after treatment with WNK463. **(F)** Nude mice were subcutaneously inoculated with OCI-AML3 cells to establish AML xenograft model and treated with WNK463, tumor volume was monitored every two days. (N = 4). **(G)** Representative images of OCI-AML3 cell xenograft tumors from the treated group and their respective controls after a 10-day treatment period. Tumor weights were summarized in statistical histograms (N = 4). **(H)** Body weights of vehicle group and WNK463 group were measured every day. *, *P* < 0.05; **, *P* < 0.01; ***, *P* < 0.001; ****, *P* < 0.0001.

To explore the role of WNK1 in AML cell survival, we tested two reported WNK inhibitors, WNK463 and WNK11 in three AML cell lines. WNK1 inhibition significantly reduced cell proliferation in all AML cell lines tested in a dose dependent manner (**Figures 2A, B**). To further assess the inhibitory effects of WNK inhibitors in AML patients, we isolated and obtained primary AML blasts from six patients covering a wider spectrum of genetic subtypes and treated the cells with WNK inhibitors for 48 h. WNK1 inhibition effectively reduced cell viability in all six primary AML cells in a dose dependent manner, with FLT3-ITD-mutated AML being particularly sensitive to WNK1 inhibition (**Figure 2C**). To determine whether WNK1 inhibition can induce apoptosis in AML cells, we performed cell apoptosis assay by flow cytometry. The result showed that WNK463 increased 30% apoptotic cells after 48h treatment (**Figure 2D**). To elucidate the mechanism of cell apoptosis induced by WNK inhibition, we treated MV4–11 cells with WNK463 for 24 hours and performed western blot. The result showed that pro-apoptotic protein Bim and Puma were upregulated after WNK inhibition, while no change were detected in anti-apoptotic proteins in both MV4–11 and THP-1 cells (**Figure 2E**). This result indicated that WNK inhibition promoted cell apoptosis but did not affect anti-apoptotic proteins. To further investigate the necessity of WNK1 for AML progression *in vivo*, we generated cell line-derived mouse xenografts of OCI-AML3 and treated the mice with WNK463 for 10 days (**Figure 2F**). In this xenograft model, WNK463 significantly reduced tumor growth consistent with the reduction of cell survival *in vitro* (**Figure 2G**), while no body weight reduction was observed (**Figure 2H**). In conclusion, these results indicated WNK1 inhibition was an effective and safe therapeutic strategy for AML.

### 3.3 WNK1 inhibition suppressed DNA replication in AML

**Figure 3:**
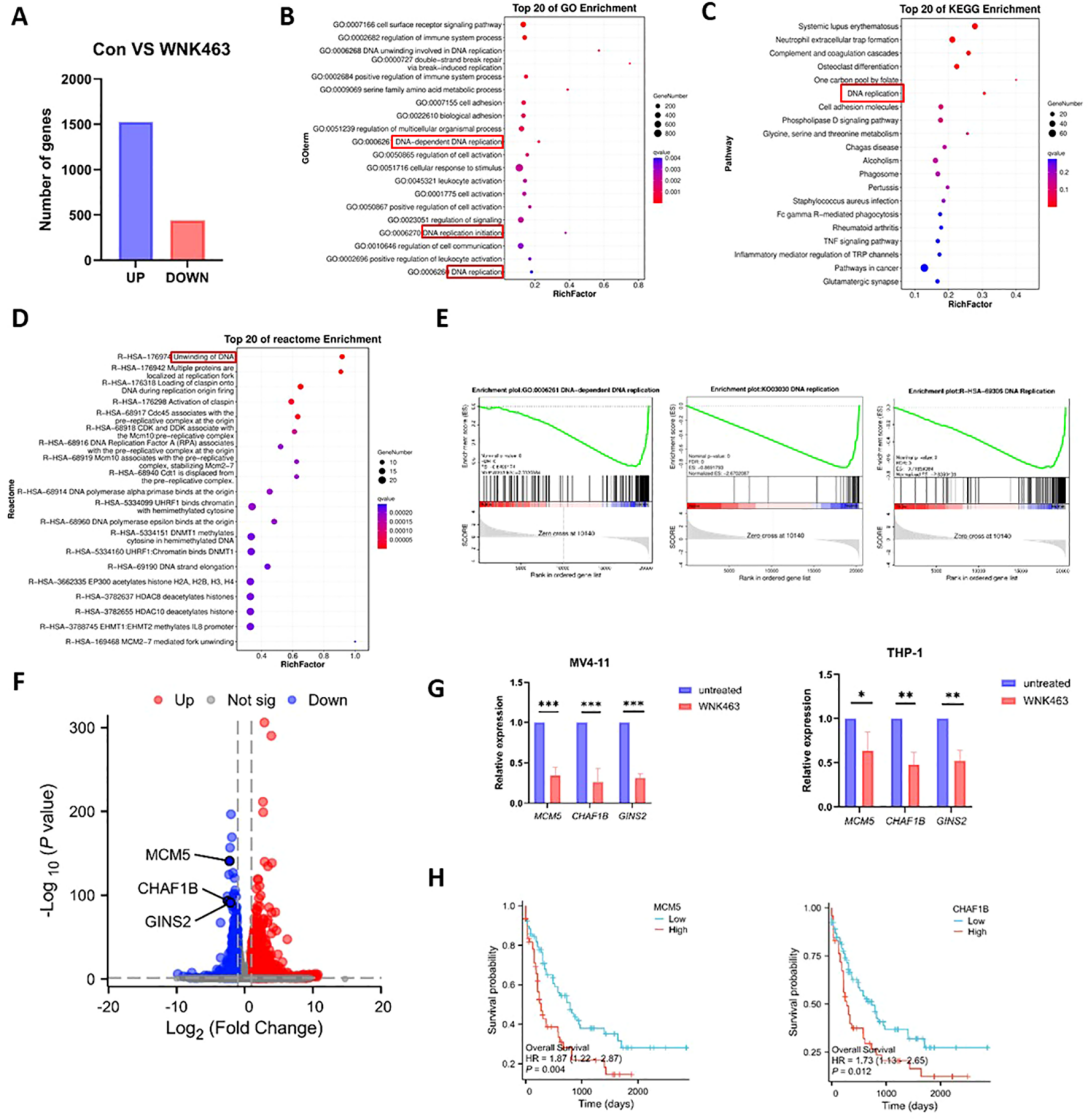
WNK1 inhibition regulates DNA replication pathways in AML. **(A)** Change of gene expression detected by RNA-seq analysis after WNK463 treatment in MV4–11 cells. **(B-D)** Gene ontology (GO) analysis **(B)**, KEGG pathway analysis **(C)**, and Reactome pathway analysis **(D)** were performed on both the control and WNK463-treated groups to explore the functional significance of the differentially expressed genes (DEGs). **(E)** Gene set enrichment analysis (GSEA) was performed in the control and WNK463-treated groups to assess differentially enriched functional gene sets. **(F)** Volcano plot illustrating the gene distribution detected by RNA-seq after WNK463 treatment in MV4–11 cells. **(G)** The expression of the MCM5, CHAF1B and GINS2 genes were evaluated by qPCR after WNK463 treatment in both MV4–11 and THP-1 cells. **(H)** Kaplan-Meier survival analysis of MCM5 and CHAF1B expression in TCGA databases. *, *P* < 0.05; **, *P* < 0.01; ***, *P* < 0.001; ****, *P* < 0.0001.

To elucidate the potential molecular mechanisms underlying the effective inhibitory effect on AML cells by WNK463, we treated MV4–11 cells with 1 μM of WNK463 or blank control for 48 h, and extracted RNA from these cells for high-throughput transcriptome sequencing. The result revealed a total of 1968 differentially expressed genes, including 1528 upregulated genes and 440 downregulated genes (**Figure 3A**). We performed GO, KEGG and Reactome analysis on the RNA-seq data and found genes were mainly enriched in DNA replication related pathways (**Figures 3B–D**). Additionally, we utilized gene set enrichment analysis (GSEA) tools to analyze all RNA sequencing data. Compared to the control group, WNK463 treated MV4–11 cells showed significant set of genes that were involved in DNA replication, which was consistent with the results from GO, KEGG and Reactome analysis (**Figure 3E**). Therefore, we focused on the genes that regulate DNA replication pathway. The volcano plot showed DNA replication related genes *MCM5, CHAF1B, GINS2* were significantly down-regulated by WNK463 in MV4–11 cells (**Figure 3F**). We performed qPCR and confirmed that the expression of *MCM5, CHAF1B, GINS2* genes was downregulated by WNK463 in both MV4–11 and THP-1 cells (**Figure 3G**). Interestingly, high expression of *MCM5* and *CHAF1B* indicated poor prognosis in AML patients (**Figure 3H**). Collectively, these results demonstrate that WNK463 induces cell apoptosis in AML cells by altering genes related to DNA replication pathways.

### 3.4 WNK1 inhibition sensitized AML cells to venetoclax

**Figure 4:**
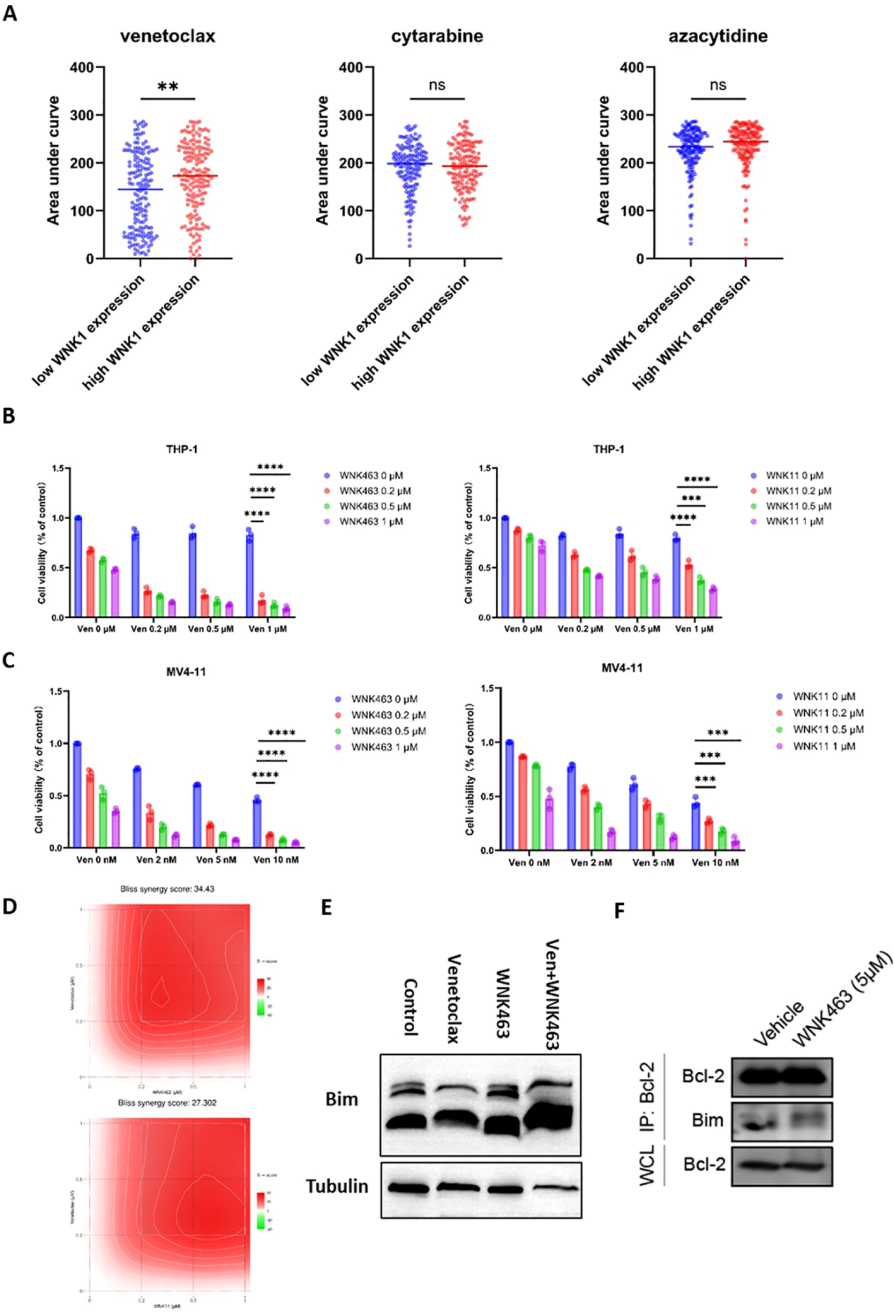
WNK1 inhibition enhanced the sensitivity of AML cells to venetoclax. **(A)** Correlation between WNK1 expression levels and AUC of venetoclax, cytarabine and azacytidine in AML. **(B, C)** Cell viabilities of THP-1 **(B)** or MV4-11 **(C)** after combination therapy of WNK1 and venetoclax for 48h. **(D)** Bliss synergy scores were calculated for venetoclax+WNK463 and venetoclax+WNK11 by SynergyFinder. **(E)** Western blot analysis was performed to detect the expression of anti-apoptotic and pro-apoptotic protein in THP-1 cells after treatment with WNK463, venetoclax and combination therapy for 24h. **(F)** Co-immunoprecipitation (Co−IP) assays were performed to determine whether WNK1 directly influences the physical interaction between BCL-2 and Bim. *, *P* < 0.05; **, *P* < 0.01; ***, *P* < 0.001; ****, *P* < 0.0001.

Based on our findings, WNK1 may be a promising therapeutic target for AML. Therefore, we were wondering if WNK1 inhibition can sensitize AML cells to current first-line therapies. We evaluated the correlation between inhibitory efficiencies and WNK1 expression by analyzing BeatAML2.0 database. AML patients with high WNK1 expression showed resistance to venetoclax, while WNK1 expression level did not affect the inhibitory effects of cytarabine or azacytidine (**Figure 4A**). Therefore, we tested whether WNK1 inhibition can sensitize THP-1 and MV4–11 cells to venetoclax. Combination of WNK inhibitors and venetoclax induced greater growth inhibition than either agent alone (**Figures 4B, C**), with Bliss synergy scores of 34.43 and 27.302 (**Figure 4D**), demonstrating synergistic effects on AML cells. Western blot analysis showed that the combination of WNK463 and venetoclax increased Bim expression compared with each drug alone (**Figure 4E**). Co-immunoprecipitation experiments further confirmed that BCL-2 directly binds Bim under physiological conditions, whereas WNK463 treatment dissociated Bim from BCL-2 (**Figure 4F**). These dual effects of WNK inhibitors synergistically induced apoptosis in AML cells. Together, WNK inhibitors exhibited strong synergy with venetoclax through upregulating Bim and promoting its dissociation from BCL-2.

## 4 Discussion

In this study, we systematically elucidated the critical role of WNK1 in acute myeloid leukemia and its potential as a therapeutic target through integrated multi-omics data analysis combined with *in vitro* and *in vivo* functional experiments. Using multiple independent public databases and samples from our hospital, we consistently demonstrated that WNK1 expression is significantly elevated in AML cell lines and patient primary cells, while the expression of other WNK family members was relatively low, suggesting that WNK1 may have a unique and non-redundant function in AML. Moreover, the high expression of WNK1 is associated with adverse AML subtypes, such as AML with FLT3-ITD mutation. Crucially, Chronos scores based on CRISPR loss-of-function screening data indicate that WNK1 is an essential gene for AML cell survival. These findings collectively establish the potential oncogenic role of WNK1 in AML pathogenesis and progression, where its elevated expression may drive disease advancement by conferring a survival advantage to the cells. Functional experiments confirmed that targeted inhibition of WNK1 effectively suppressed the proliferation of AML cell lines and primary cells, induces apoptosis, and significantly inhibits tumor growth in a mouse xenograft model without causing significant weight loss. This preliminarily demonstrated the anti-leukemic activity and favorable tolerability of WNK1 inhibitors. Our findings underscore the importance of WNK1 as a therapeutic target for AML. However, the functional mechanism of WNK1 is distinctly different in multiple myeloma. WNK1 has been shown to influence the expression of MYC and other IgH translocation partner genes by modulating IgH enhancer activity. Both genetic and pharmacological inhibition of WNK1 reduced MYC expression, thereby suppressing MM cell growth *in vitro* and *in vivo* (12). Furthermore, single-cell sequencing analyses reveal that WNK1 is closely associated with a key subpopulation of malignant proliferating plasma cells in MM, characterized by robust proliferative capacity, high copy number variation (CNV) scores, and active glycolytic metabolism (14). Collectively, these findings suggest that WNK1 functions predominantly as a transcriptional-epigenetic regulator in MM. In chronic lymphocytic leukemia (CLL), the role of WNK1 has been elucidated through an integrated single-cell genetic and transcriptomic approach. By performing targeted mutation detection in thousands of single cells from five CLL samples, Wang and colleagues identified WNK1 mutations as a novel driver event in CLL, with functional evidence indicating a significant impact of these mutations on CLL signaling pathways (11). Notably, WNK1 is recognized as a driver in CLL primarily based on its somatic mutation status, rather than through functional upregulation of kinase activity as observed in AML or MM. Collectively, these reports broaden the spectrum of WNK1’s roles in hematologic malignancies.

Transcriptomic analysis suggests that WNK1 inhibition leads to significant downregulation of gene sets related to DNA replication pathways, including MCM5, CHAF1B and GINS2. MCM5, one of the key DNA replication licensing factors, was downregulated by WNK463. As a key component of the MCM2–7 core helicase complex, MCM5 is essential for the initiation of DNA replication. In tumor cells, the overexpression of MCM5 often leads to uncontrolled DNA replication and an abnormal acceleration of the cell cycle, thereby promoting the unlimited proliferation of tumor cells (15, 16). Moreover, Mcm5 knockout upregulated p53 signaling genes including Bbc3 in immature T lymphocytes, thereby establishing a connection between replication stress and apoptosis (17). Our results also showed high expression of MCM5 is associated with poor prognosis in AML patients. These results imply that WNK1 may maintain the highly proliferative state of AML cells by regulating the expression of DNA replication-related genes. Inhibiting WNK1 could disrupt this process, potentially inducing replication stress and ultimately leading to cell apoptosis, which aligns with our observation of upregulation of the pro-apoptotic proteins Bim and Puma.

Venetoclax, as a selective BCL-2 inhibitor, plays a revolutionary role in the treatment of AML. For elderly AML patients who are unsuitable for intensive chemotherapy, venetoclax combined with hypomethylating agents or low-dose cytarabine has become the standard treatment regimen, significantly improving patients’ response rates and overall survival (17). Despite the substantial clinical benefits offered by venetoclax, most patients ultimately develop resistance and experience disease relapse, which remains a major challenge in AML treatment (18). Therefore, one of the most translational findings of this study is the synergistic effect between WNK1 inhibition and venetoclax. Database analysis revealed that high WNK1 expression correlates with venetoclax resistance, suggesting WNK1 as a potential biomarker and intervention target for resistance. *In vitro* experiments confirmed that the combination of WNK inhibitors and venetoclax produces a significant synergistic anti-proliferative effect, accompanied by further upregulation of the pro-apoptotic protein Bim. As shown in previous research, when the expression level of Bim protein in cells is high, Bim can bind to BCL-2 more effectively. Venetoclax competitively binds to BCL-2, further releasing Bim protein and allowing it to act on Bax/Bak and accelerate the process of cell apoptosis. Therefore, high Bim expression is typically associated with greater sensitivity of AML cells to venetoclax (19, 20). Moreover, co-immunoprecipitation experiments confirmed that WNK463 treatment further dissociated Bim from BCL-2, revealing a novel mechanism by which WNK inhibitors synergize with venetoclax: the simultaneous upregulation of Bim and facilitation of its release from BCL-2.

In summary, this study systematically demonstrates that WNK1 is a novel therapeutic target in AML. Its inhibitors exert anti-leukemic effects by interfering with critical processes such as DNA replication and can synergize with venetoclax. These results lay a solid experimental foundation for developing novel therapies or combination regimens targeting WNK1, potentially offering new treatment options for AML patients, especially those at risk of venetoclax resistance. Future research directions should include developing more selective WNK1 inhibitors, validating their efficacy in a broader range of AML genetic subtypes, and deeply exploring the role of WNK1 in AML stem cell maintenance and drug resistance.

## 5 Conclusions

In conclusion, our study establishes WNK1 as a critical oncogenic kinase in AML pathogenesis and a promising therapeutic target. We demonstrate that WNK1 is selectively overexpressed in AML and essential for leukemia cell survival. Pharmacologic inhibition of WNK1 effectively suppresses AML progression both *in vitro* and *in vivo* by inducing apoptosis and disrupting DNA replication pathways, as evidenced by downregulation of key regulators such as MCM5 and CHAF1B. Notably, we identify a novel synergy between WNK1 inhibition and venetoclax, linking high WNK1 expression to venetoclax resistance and showing that combined targeting enhances pro-apoptotic Bim upregulation and anti-leukemic efficacy. These findings not only elucidate the functional role of WNK1 in AML but also provide a strong preclinical rationale for developing WNK1-targeted therapies, either as monotherapy or in rational combinations with venetoclax, to overcome resistance and improve outcomes for AML patients. Future studies should focus on advancing selective WNK1 inhibitors and validating this strategy across diverse genetic subtypes and in leukemic stem cell populations.

## Data Availability

The data presented in the study are deposited in the NCBI GEO repository, accession number GSE333251.
